# Tumor Lysis Syndrome Caused by Unrecognized Richter’s Transformation of Chronic Lymphocytic Leukemia: Treatment With Venetoclax for Suspected Disease Progression

**DOI:** 10.7759/cureus.8145

**Published:** 2020-05-15

**Authors:** Juan Jose Chango Azanza, Paola Michelle Calle Sarmiento, Vinay Mathew Thomas, Nerea Lopetegui Lia, Neiha Kidwai

**Affiliations:** 1 Internal Medicine, University of Connecticut Health Center, Farmington, USA; 2 Internal Medicine, Catholic University of Cuenca, Cuenca, ECU

**Keywords:** tumor lysis syndrome, venetoclax, richters transformation, chronic lymphocytic leukemia, diffuse large b cell lymphoma

## Abstract

Richter’s transformation (RT) is defined as the transition of chronic lymphocytic leukemia (CLL) or small lymphocytic leukemia (SLL) into an aggressive lymphoma. The conversion generally leads to diffuse large B-cell lymphoma (DLBCL), but more aggressive forms such as Hodgkin lymphoma (HL) can also occur. RT is a rare complication of CLL. RT can be confused with CLL progression. Its identification is crucial because the management of lymphoma and CLL differ from each other. Furthermore, the use of certain agents for CLL such as venetoclax increases the risk of tumor lysis syndrome (TLS) in neoplasms with rapid replication such as DLBCL or CLL with hyperleukocytosis (blast crisis). We present the case of a 76-year-old man with a history of CLL on chemotherapy who developed fatigue, malaise, night sweats, chills, and unintentional weight loss for which he was started on treatment with venetoclax due to suspected clinical progression of his disease. The patient developed TLS, requiring hospitalization, and he was found to have an acute blast crisis. Also, his CLL was found to have been transformed into an aggressive DLBCL. This case highlights the importance of differentiating a true progression of CLL from RT into an aggressive lymphoma given that treatment would be different for the two and the prognosis with the transformation is worse.

## Introduction

Chronic lymphocytic leukemia (CLL) is a common hematologic malignancy. Richter’s transformation (RT) or syndrome is defined as the transition of CLL or small lymphocytic leukemia (SLL) into an aggressive lymphoma such as diffuse large B-cell lymphoma (DLBCL) or Hodgkin lymphoma (HL) [1]. RT is a rare complication of CLL, which occurs in approximately 2-10% of patients with CLL. The transformation rate is approximately 0.5-1% per year [2]. It might be challenging to differentiate when patients are undergoing a blast crisis or hyperleukocytosis versus a transformation into a lymphoma (RT). The clinical features to suspect that a patient may be experiencing a transformation are a marked increase in lymphadenopathy at one or more sites, splenomegaly, or increased "B" symptoms characterized as fevers, night sweats, and weight loss. Lactate dehydrogenase (LDH) elevation is another useful marker. Worsening anemia and thrombocytopenia can also be seen [3]. The management of CLL and DLBCL or HL differ from each other. Therefore, it is crucial to identify RT when it occurs as there are important implications in its management, complications, and prognosis. We present the case of a patient who developed a suspected progression of CLL for which he was treated with venetoclax; he went on to develop tumor lysis syndrome (TLS). He was found to have hyperleukocytosis and RT into a DLBCL.

## Case presentation

A 76-year-old man with a history of a B-cell CLL presented to his oncologist's office for a follow-up of laboratory results. He endorsed having fatigue and generalized malaise that had significantly worsened in the last three days. He had been experiencing night sweats, chills, and unintentional weight loss of 8-10 pounds for the last three months. The last visit to his oncologist had been four days prior, and he had been started on venetoclax (a BCL-2 or B-cell lymphoma 2 inhibitor) due to suspicion of clinical progression of his disease. His oncologist noted abnormal laboratory results and referred him to the emergency department. In the hospital, his vital signs were within normal limits and no major abnormalities other than signs of dehydration were appreciated on physical examination. His past medical history was significant for a B-cell CLL diagnosed nine years prior. He had been treated with chlorambucil initially and then bendamustine, rituximab, and ibrutinib for two years. Other relevant past medical history included hypogammaglobulinemia treated with intravenous immunoglobulin infusions every month.

**Figure 1:**
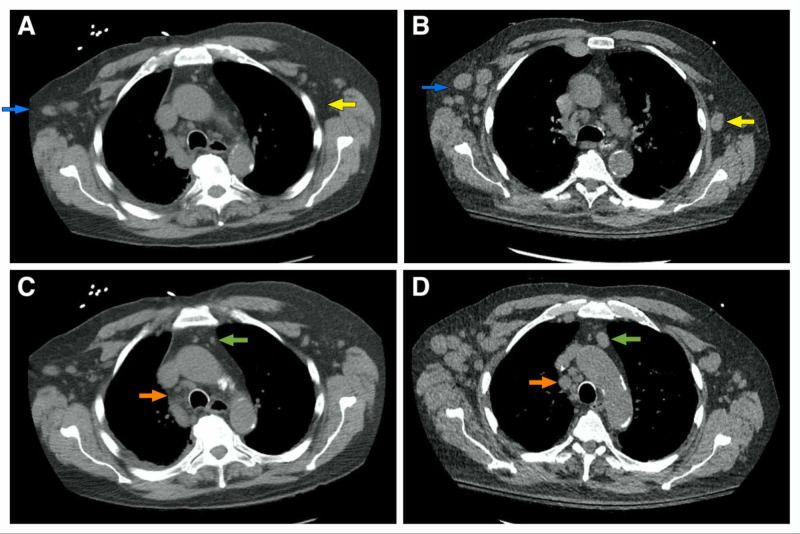
CT scan of the chest Increased bilateral axillary adenopathies (blue and yellow arrows) are smaller in the previous CT scan done 10 months prior (A) when compared to the new scan (B). Similarly, increased mediastinal adenopathies are evident (green and orange arrows) when comparing an old scan (C) with the newer study (D) CT: computed tomography

**Figure 2:**
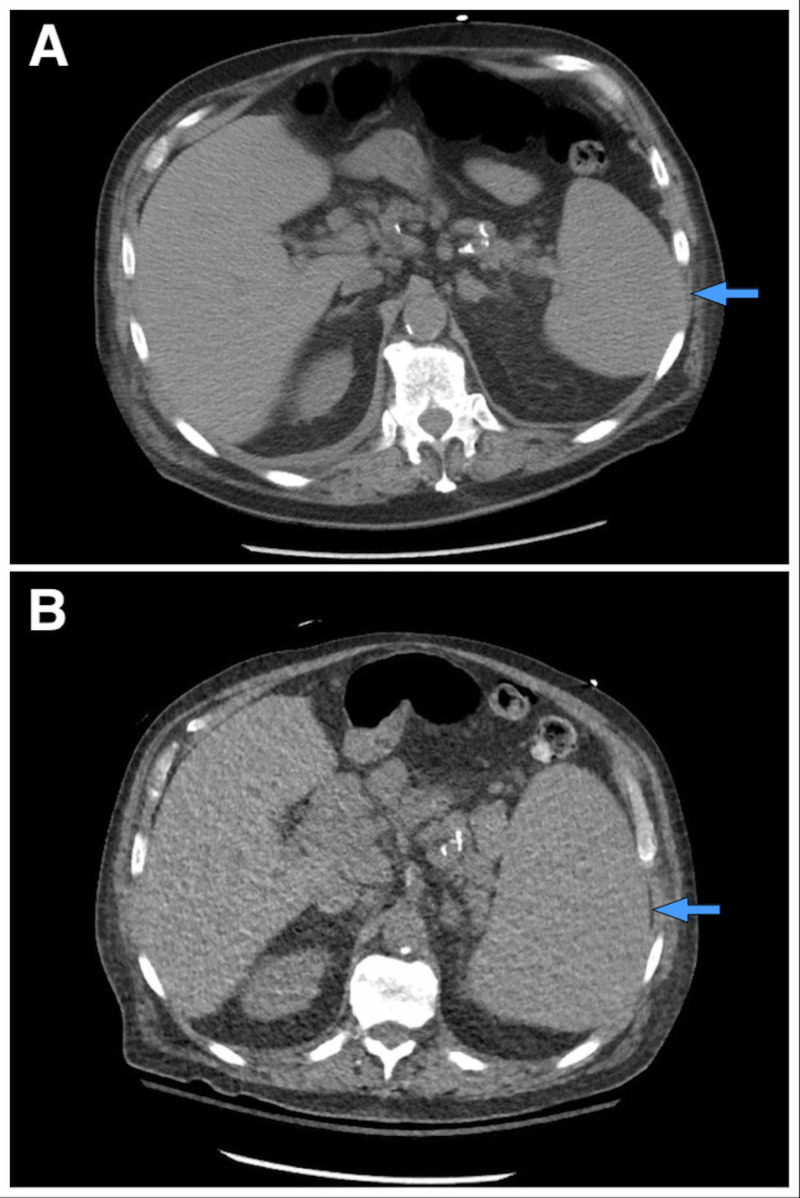
CT scan of the abdomen The image shows a normal-sized spleen (blue arrows) 10 months prior (A) in comparison to newly-found prominent splenomegaly (B) during his current hospitalization CT: computed tomography

Initial laboratory workup including a complete blood count showed a white blood cell (WBC) count of 164,600/mm^3^ (with a baseline WBC of 14,700/mm^3^), hemoglobin of 9.5 g/dL, hematocrit of 29.2%, mean corpuscular volume (MCV) of 95 um^3^, and platelet count of 102,000/mm^3^. The WBC differential showed 8% neutrophils (14.8 cells/mm^3^), 88% of lymphocytes (144.8 cells/mm^3^), 1% monocytes, 1% basophils, 1% bands, and 1% myelocytes. His chemistry showed a potassium level of 8.6 mEq/L, creatinine of 3.5 mg/dL, calcium of 9.0 mg/dL, phosphate of 3.7 mg/dL, uric acid of 26.4 mg/dL, and an LDH level of 6,861 U/L. His electrocardiogram did not show any abnormalities. A peripheral blood smear demonstrated increased prolymphocytes, anemia, and thrombocytopenia with no macrothrombocytes or spherocytes. A CT scan of the chest done 10 months prior had not shown relevant axillary adenopathies (Figure 1A). However, new diffuse axillary lymphadenopathies were present in a new CT scan of the chest done during admission (Figure 1B). Furthermore, there was no prominent mediastinal adenopathy in the last CT scan of the chest (Figure 1C), but now he also developed worsening mediastinal adenopathies (Figure 1D). His spleen had not been enlarged in previous CT scans (Figure 2A), but now he experienced new significant splenomegaly (Figure 2B). He was admitted to the hospital with a high suspicion of TLS in the setting of a blast crisis and venetoclax use.

**Figure 3:**
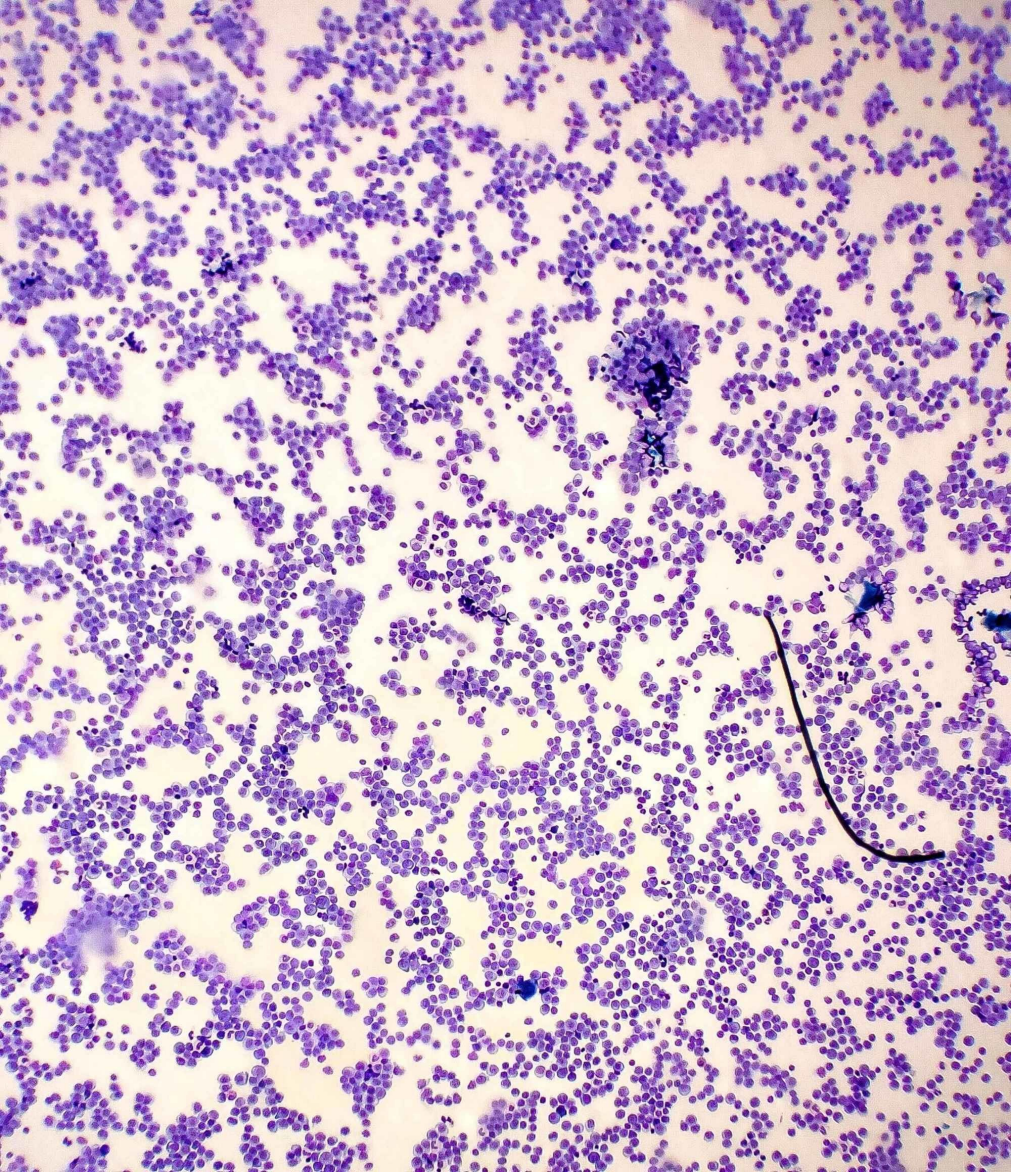
Low-power electronic microscopy showing marked lymphocytosis

**Figure 4:**
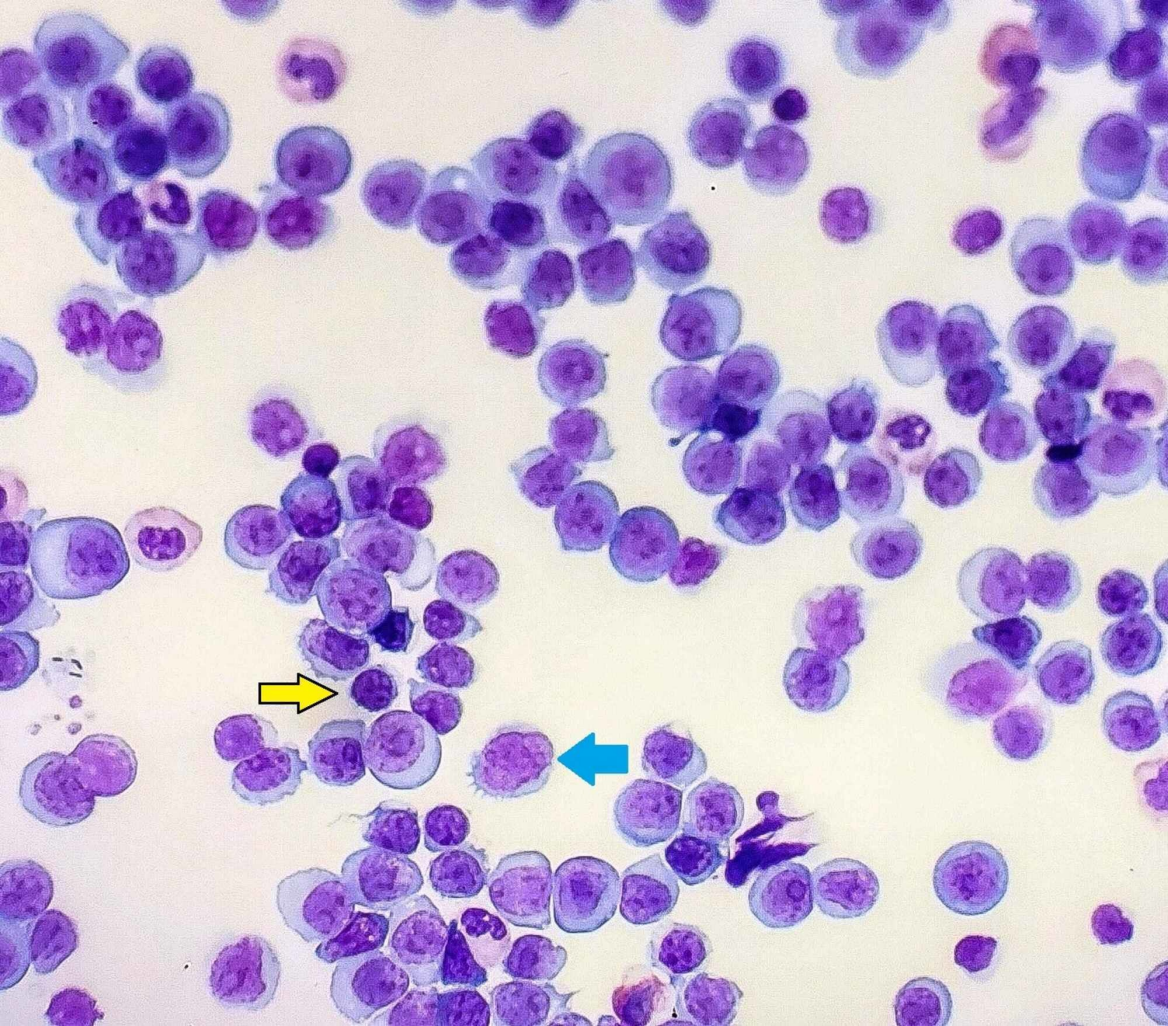
High-power electronic microscopy The image shows abnormal lymphoid cells with medium to large-sized ovoid nuclei, delicate chromatin, prominent nucleoli, and increased basophilic cytoplasm (blue arrow). A difference in cell size can be appreciated when comparing the normal-sized lymphocytes (yellow arrow) to the pathologic lymphoid cells. This phenomenon termed "Richters transformation" demonstrates the shift from normal-sized lymphocytes seen in chronic lymphoblastic leukemia into larger cells observed in diffuse large B-cell lymphoma

Furthermore, a flow cytometry analysis of his peripheral blood was performed but was not readily available. The repeat microscopy showed a WBC count of 250,000/mm^3^ with marked lymphocytosis of abnormal medium to large-sized lymphoid cells (Figure 3). These lymphocytes were characterized by an ovoid nucleus, prominent nucleoli, delicate chromatin, and increased basophilic cytoplasm (Figure 4). By flow cytometry and cytogenetic analysis, it was found that 95% of WBCs were abnormal B cells with intermediate forward scatter and mildly increased side scatter for CD5, CD19, CD20 (moderate), CD22, CD23, CD45, FMC7, and kappa restriction. There was a dim partial expression of CD11c and CD25. No significant CD10 or CD103 expression was observed. More than 90% of CD19/CD5 coexpressed B cells displayed CD38. These findings depicted markedly increased levels of monoclonal CD5+ B cells with profound morphologic atypia. The immunophenotype was not characteristic of a B-cell CLL, thus depicting a transformation to an aggressive large B cell lymphoma - a phenomenon termed RT.

The patient was administered intravenous (IV) fluids and rasburicase. His hyperkalemia was immediately treated with insulin and dextrose, calcium, patiromer, and a low-potassium diet. Oncology recommended starting allopurinol after 72 hours of admission. The decision to start chemotherapy in the setting of a blast crisis was considered but complicated by TLS. The metabolic disturbances and kidney injury improved with IV fluids and the management of electrolyte disturbances. Uric acid decreased after rasburicase administration. Unfortunately, his WBC count increased without chemotherapy to 258,000/uL within the first four days of his hospital stay. Due to the aggressive nature of his disease with an adverse prognosis and progression of a new DLBCL, the patient and his family decided not to pursue restorative treatment. The patient was transitioned to a comfort care approach. No further diagnostic workup or treatment was pursued. The patient expired a few days later.

## Discussion

CLL is a common form of leukemia in adults. RT is defined as the transition from a low-grade lymphoproliferative disorder such as CLL or SLL into an aggressive lymphoma. The median time from diagnosis of the low-grade B cell malignancy to the transformation into large B cell lymphoma is two to four years [4]. The risk factors for RT differ from CLL risk factors. Certain features increase the risk for RT, such as clinical (Binet stage B/C, lymphadenopathy, performance status), biochemical (LDH elevation), biological [expression of CD38 and ZAP70, unmutated immunoglobulin heavy chain variable gene (IGHV)], and cytogenetic (del(13q) absence, (tri12), del(11q) and del(17p), TP53, NOTCH1, CDKN2A, c-MYC activation) [5]. Age of >65 years and male sex are risk factors for RT. Other risk factors include CLL-treatment regimens such as purine-nucleoside analog and/or alkylating agents plus/minus monoclonal antibody therapy and/or kinase inhibitor therapy, radiation therapy, and stem cell transplantation. Some other treatments have also been hypothesized to be responsible as well [6].

It might be challenging to differentiate when patients are undergoing a blast crisis or hyperleukocytosis versus a transformation into a lymphoma (RT). There are several clinical features to suspect RT, including a marked increase in lymphadenopathy at one or more sites, splenomegaly, or increased "B" symptoms identified as fevers, night sweats, and weight loss. LDH elevation is also typically found [7]. Worsening anemia and thrombocytopenia can be seen [3]. Less frequently, RT can be associated with extranodal involvement of the central nervous system (CNS), testes, eyes, and lungs [8]. Most patients that develop RT have a history of CLL. However, RT can also be the first presentation of the disease, which is sometimes referred to as "*de novo* Richter’s transformation." It might be important to consider RT as the cause of spontaneous TLS in patients with a history of CLL. The clinical features encountered in RT can also be seen due to progressive CLL, especially after the acquisition of del(17p13.1) or TP53 inactivation [2].

Laboratory findings in RT such as lymphocytosis, neutropenia, anemia, and thrombocytopenia are also seen in CLL. However, LDH elevation (seen in 82% of patients) and/or monoclonal gammopathy (found in 44% of patients) can be important clues for RT [2]. Peripheral blood smears can show atypical large cells with scant cytoplasm and distinct nucleoli [9]. RT cells are usually larger than CLL cells. Flow cytometry and cytogenetic studies using different techniques with analysis of CD62L and CD52, karyotype, MYC abnormalities by fluorescence in situ hybridization (FISH), and other studies are helpful in the differential diagnosis. Loss of expression of CD52 in RT most likely predicts resistance to alemtuzumab, one of the most frequently used therapeutic agents for CLL [10]. Tissue analysis is important in making a definitive diagnosis. The site selection for biopsy and pathologic sample collection is an important step. There are different imaging modalities that can aid in the diagnosis as well as biopsy selection site determination. CT and positron emission tomography (PET) scans are used in the evaluation of the suspected transformation and to select the biopsy site. When using PET/CT scans with a standardized uptake value (SUV) threshold of 5 as a cutoff, the positive predictive value is 53% and negative predictive value is 97% for RT [11]. Having a high negative predictive value reduces the post-test probability of RT in patients without a fluorine 18 fluorodeoxyglucose (FDG) avid lymphadenopathy. The low positive predictive value of PET/CT means that the selection of the biopsy site is crucial to increase the diagnostic yield. Only half of the patients with a tissue biopsy will have a positive result for RT [2]. The other causes of FDG-avid lymphadenopathy are CLL progression, inflammatory conditions, and infections. Once a tissue diagnosis is achieved, the next step is to perform a bone marrow biopsy for staging purposes. FISH studies should be performed in peripheral blood and the bone marrow samples for the identification of del(17p13.1) because this genetic finding has implications in treatment selection [2].

Regarding treatment, one of the most important steps is to determine if the DLBCL is clonally related to the underlying CLL. In RT, 80% of DLBCL are clonally related to the underlying CLL while 20% are not [2]. When patients have clonally unrelated DLBCL, treatment would be similar to the *de novo* DLBCL with rituximab, cyclophosphamide, doxorubicin, vincristine, and prednisone (R-CHOP). When patients achieve complete remission (CR), no further treatment is needed, and periodic surveillance is indicated. However, when no CR is reached after R-CHOP therapy, salvage therapy with rituximab, ifosfamide, and etoposide (RICE) or rituximab, dexamethasone, cytarabine, and cisplatin (RDHAP) followed by stem cell transplantation should be considered [12]. For the clonally related DLBCL, standard treatment approaches are usually suboptimal [2]. Therefore, treatment should generally be a clinical trial if available. If a clinical trial is not feasible, the recommendation is to consider R-CHOP as we would do for a *de novo *DLBCL. When patients have received anthracycline therapy, a platinum-based regimen is preferable. Stem cell transplant is another option for selected patients depending on functional status, age, comorbidities, and chemotherapy sensitivity [2].

## Conclusions

RT is a known complication of CLL/SLL in which an aggressive lymphoma such as a DLBCL arises. When monitoring patients with CCL/SLL, it is important to keep in mind that the progression of CLL can be confused with RT. Treatment of CLL differs from that of DLBCL and hence it is imperative to recognize RT given the implications in management, prognosis, and complications. RT carries a worse prognosis. Before starting treatment with certain agents such as venetoclax, it is crucial to identify RT and the tumor burden to prevent complications such as TLS because DLBCL has a higher risk for lysis than CLL due to rapid replication and tumor bulk. TLS was identified in our case, which was not as expected for CLL as it would have been for DLBCL.
